# Hepatic mitochondrial dysfunction in Friedreich Ataxia

**DOI:** 10.1186/1471-2377-11-145

**Published:** 2011-11-15

**Authors:** Sven H Stüwe, Oliver Goetze, Larissa Arning, Matthias Banasch, Wolfgang E Schmidt, Ludger Schöls, Carsten Saft

**Affiliations:** 1 Department of Neurology, Ruhr-University, St. Josef-Hospital, Bochum, Germany; 2 Department of Internal Medicine I, Ruhr-University, St. Josef-Hospital, Bochum, Germany; 3 Division of Gastroenterology and Hepatology, University Hospital Zurich, Switzerland; 4 Department of Human Genetics, Ruhr-University Bochum, Germany; 5 Department of Neurology and Hertie Institute for Clinical Brain Research, Tübingen, Germany; 6 German Center for Neurodegenerative Diseases (DZNE), Tübingen, Germany

**Keywords:** ^13^C-methionine, breath test, Friedreich, Ataxia, neurodegeneration

## Abstract

**Background:**

Mitochondrial dysfunction due to respiratory chain impairment is a key feature in pathogenesis of Friedreich ataxia. Friedreich ataxia affects the nervous system, heart and pancreas.

**Methods:**

We assessed hepatic mitochondrial function by ^13^C-methionine-breath-test in 16 Friedreich ataxia patients and matched healthy controls.

**Results:**

Patients exhaled significantly smaller amounts of ^13^CO_2 _over 90 minutes. Maximal exhaled percentage dose of ^13^CO_2 _recovery was reduced compared to controls.

**Conclusions:**

^13^C-methionine-breath-test indicates subclinical hepatic mitochondrial dysfunction in Friedreich ataxia but did not correlate with GAA repeat lengths, disease duration or disease severity.

## 1. Background

Friedreich ataxia (FRDA) is an autosomal recessive neurodegenerative disorder caused by expanded GAA triplet repeats located in the first intron of the *FXN *gene coding for *frataxin *on chromosome 9 [1]. Frataxin is a mitochondrial protein involved in biogenesis of iron-sulfur clusters (ISCs). ISCs serve as prosthetic group in several enzymes of the mitochondrial energy metabolism including aconitase and complexes I, II and III of the respiratory chain that are impaired in FRDA [2]. Frataxin mRNA was found to show a broad expression pattern, including tissues with a high metabolic rate, like liver, kidney, brown fat and heart [3]. In addition to mitochondrial dysfunction, increase of reactive oxygen species (ROS)s is regarded as key feature in FRDA pathogenesis [4]. The length of the shorter of the two GAA repeats is thought to determine the residual amount of frataxin and thereby influencing age at onset of symptoms and disease severity [5,6]. Additionally, repeat length influences the development of diabetes and cardiomyopathy [5].

Several clinical rating scales have been proposed to assess disease severity in FRDA. The "scale for the assessment and rating of ataxia" (SARA) was evalutated in FRDA with high interrater reliability and practicability [7]. For future trials, especially for a possible neuroprotective treatment, there is a need for biomarkers linked to pathogenesis of FRDA. Beside others, quantification of frataxin, proton magnetic resonance spectroscopy (MRS) of the brain and phosphorus MRS in calf muscle [8] as well as atrophy of spinal cord and the medulla [9] were described as potential biomarkers in FRDA.

Methionine, an essential amino acid, is mainly metabolized in the liver which is remarkable rich in mitochondria [10]. Excess of methionine methyl groups is metabolised via sarcosine (N-methylglycine) and mitochondrial oxidation to CO_2 _[10]. Since the highest specific activity of methionine adenosyltransferase in mammals occurs in hepatic tissue, methyl-^13^C-methionine breath test (MeBT) has been proposed for the assessment of hepatic mitochondrial function *in vivo *[11]. Meanwhile MeBT is established for non-invasive, easy to perform and cheap quantification of oxidative capacity of liver mitochondria and was used for drug monitoring in HIV and Hepatitis C [12,13]. Further applications include detecting of drug and alcohol related liver toxicity, liver steatosis and cirrhosis [10,14]. The method has not been used in neurological disorders so far.

The aim of this study was to investigate mitochondrial liver function of FRDA patients and to explore if the MeBT is feasible to reflect disease severity in FRDA.

## 2. Methods

16 patients all with genetically confirmed Friedreich ataxia and homozygous for the GAA expansion were recruited from the Department of Neurology, St. Josef-Hospital, Ruhr-University Bochum and the Ataxia Clinic, Department of Neurodegeneration, University of Tübingen, Germany. Healthy volunteers were matched to patients by age, gender and body surface area (BSA; see Fischer Analysen Instrumente GmbH, Leipzig, Germany). All participants gave written informed consent to the study. The study was approved by the ethic committee of the Ruhr-University Bochum, (registration-number 2718). Exact GAA repeat lengths in the *FXN *gene were available in 14 of 16 FRDA patients. Two patients had a late onset of the disease (28 and 30 years), only one was still able to walk independently. Disease severity was assessed using SARA (table 1) [7]. Six FRDA participants were free of medication, four were on proton pump inhibitors (PPIs) and beta-blockers, two respectively on baclofen 5 or 10 mg, ACE-inhibitors, digitoxin and one respectively on buprenorphine 0.2 mg, ropinirole 4 mg, olmesartan 20 mg, trospium chloride 60 mg, etoricoxib 60 mg, clonazepam 1 mg, citalopram 10 mg, amitriptyline 50 mg, gabapentin 800 mg, prednisone 7.5 mg, hydrochlorothiazide 25 mg, acetylsalicylic acid 300 mg daily. Three FRDA patients got antioxidants; one took L-carnitine 1500 mg, coenzyme Q(10) 400 mg and vitamine E 1000 mg, one idebenone 2250 mg and one L-carnitine 1500 mg. All participants paused with their medication for at least 12 hours prior to the MeBT. Exclusion criteria were concurrent liver diseases, excessive alcohol consumption (50 g/d of ethanol), severe other diseases, underage and pregnancy.

**Table 1:** Baseline group statistics of FRDA patients and healthy controls

Parameter	FRDA (n = 16)	controls (n = 16)
Age [yr]	43.8 ± 9.3 (28-59)	43.1 ± 8.7 (28-60)
Gender (male/female)	10/6	10/6
Height [m]	1.72 ± 0.1 (1.59-1.83)	1.73 ± 0,1 (1.57-1.89)
Weight [kg]	73.9 ± 9.9 (58-95)	74.8 ± 9 (56-94)
BSA [m^2^]	1.89 ± 0,2 (1.61-2.20)	1.9 ± 0.2 (1.61-2.16)
GAA shorter allel *	402 ± 127 (220-560)	--
GAA larger allel *	798.5 ± 250.6 (250-1110)	--
GAA product both alleles *	394492.9 ± 245596.8 (62500-954600)	--
Onset of the disease [yr]	17.2 ± 5.9 (7-30)	--
Disease duration [yr]	26.7 ± 8.7 (8-42)	--
SARA	26.5 ± 7 (9.5-36)	--

Values are given as mean ± SD; range (min-max) in brackets; Abbreviations: BSA - body surface area, yr - years, SARA - scale for the assessment and rating of ataxia. *Exact GAA allel length was available for n = 14

The ^13^C-methionine breath test was prepared as established by Banasch *et al *2008 [15]. ^13^C-methionine breath test was run after an overnight fasting. All subjects received [methyl-^13^C]-labelled methionine (L-methionine-^13^C, 99% atom isotopic enrichment; Cambridge Isotope, Andover, MA) in a dose of 2 mg/kg of body weight dissolved in 100 ml of water. Breath samples were collected before substrate administration at baseline and then every 10 minutes for 90 minutes in 50 ml closed aluminized plastic breath bags. During the test patients were requested to sit relaxed. The collected breath samples were analysed by measuring the ^13^C/^12^C isotope ratio via nondispersive isotope selective infrared spectroscopy (IRIS; ^13^C Wagner Analysen Technik, Bremen, Germany). Results were expressed as delta (δ) ^13^C/^12^C and as delta over baseline (DOB). PDB-standard from South Carolina (Ratio_PDB _= 0.0112372) was used for the analysis of the data. The results were expressed as percentage dose of ^13^C recovery (PDR) over time for each time interval, maximum PDR (PDR_max_) and cumulative PDR (cPDR) after different minutes of testing time up to 90 min (cPDR90).

Statistical analysis was performed using the software program SPSS statistics 18. All measured parameters and clinical data are presented as mean ± SD. Normality of distribution of the data was tested by one-sample Kolmogorov-Smirnov test. For testing the significance of differences between the two groups, independent t test procedure was used. Homogeneity of variance was shown by Levene's test. For specification of relationship between ^13^C-methionine breath test results and FRDA rating scales as well as clinical data of patients a stepwise linear regression model was performed. The significance level of the F value for accepting an independent variable to the linear regression model was chosen as p = 0.05 and for exclusion as p = 0.1.

## 3. Results

Demographic, clinical and genetic characteristics of FRDA participants and matched healthy controls are provided in table 1. All variables showed normal distribution. Independent t-test for scale parameter and Fisher exact homogeneity Chi-square test for categorical parameter revealed no significant differences (t-test: p > 0.1, Chi-square test: p = 1) between the FRDA group and the control group.

### 3.1. ^13^C-Methionine Breath Test

**Figure 1:**
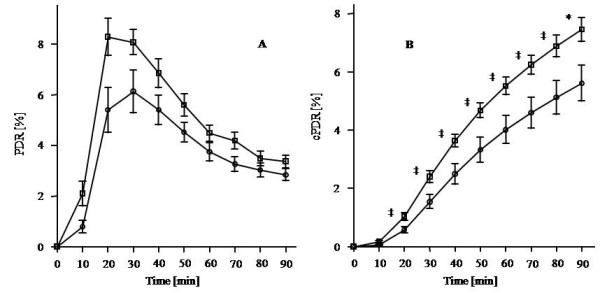
Mean recovered ^13^CO_2 _as function of time (PDR [%/h]) (A) and mean cumulative exhaled ^13^CO_2 _(cPDR [%]) (B) of 16 FRDA patients (circle) and 16 healthy controls (square); Error bares = ± SEM; * - significant difference between the groups (p < 0.05); ‡ - explorative difference between the groups (p < 0.05).

Significant decay of mitochondrial function was found in FRDA patients in comparison to controls for the cumulative percentage dose rate over 90 minutes of exhaled ^13^CO_2 _(cPDR90 [%] ± SEM: 5.61% ± 0.61% vs. 7.45% ± 0.4%; p = 0.018; Figure 1B). Additionally, the maximal exhaled percentage dose of ^13^CO_2 _recovered was reduced in the FRDA patient group (PDR_max _± SEM: 6.82%/h ± 0.81%/h vs. 9.71%/h ± 0.6%/h; p = 0.007; at 33 ± 10.5 min, see Figure 1A). In an additional explorative analysis, independent t-test was calculated for the cPDR of each time point. Here differences of cPDR between FRDA and matched controls were found already after test duration of 20 minutes (Figure 1B). No differences were observed between drug-free FRDA patients (n = 6; cPDR90 [%] ± SEM: 6.99% ± 0.67%, PDR_max _± SEM: 8.65%/h ± 0.93%/h) and patients on medication (n = 10; cPDR90 [%] ± SEM: 4.79% ± 0.8%, PDR_max _± SEM: 5.72%/h ± 1.05%/h). No differences were observed between patients with antioxidants (n = 3; cPDR90 [%] ± SEM: 5.86% ± 1.24%, PDR_max _± SEM: 6.86%/h ± 1.42%/h) and patients without antioxidants (n = 13; cPDR90 [%] ± SEM: 5.56% ± 2.69%, PDR_max _± SEM: 6.81%/h ± 3.56%/h; p = 0.855, 0.979 respectively).

Liver metabolism as assessed by cPDR and PDR_max _did not correlate with genetic markers (expanded GAA repeats and their interaction) nor with age, age at onset of symptoms or duration of disease and did not reflect disease severity as assessed by SARA using multiple linear regression analysis.

## 4. Discussion

The present cross-sectional study is the first to show subclinical liver affection in FRDA. Since participants with concurrent liver diseases or excessive alcohol consumption were excluded, none of the participants was suffering from a clinical manifest liver affection. Using the ^13^C-methionine breath test, however, we could clearly demonstrate mitochondrial dysfunction in the liver in vivo. This is in accordance with histophalogical findings, describing an increased iron deposition not only in FRDA heart, but also in liver and spleen in a pattern consistent with a mitochondrial location [16]. Down regulation or defect of citric acid cycle enzymes like aconitase, defects in complex I/II/III of the endoxidation and thus a failure of redox-equivalent oxidation necessary for the citric acid cycle are potential mechanisms. The reduced mitochondrial function in MeBT confirms the importance of frataxin for mitochondrial energy metabolism in FRDA [2].

Subclinical, impaired mitochondrial liver function should be considered when applying drugs with potential mitochondrial toxicity like valproate or propofol that are known to exert critical effects in other mitochondrial disorders like polymerase gamma (POLG) mutations [17]. One case with fatal liver failure associated with valproate therapy in a patient with FRDA has been described so far [18].

Contrary to our expectation, no correlations between MeBT and disease severity, age of onset or disease duration could be established. Although inverse correlation of clinical severity and repeat lengths has repeatedly been reported in FRDA,[5,6,19] we found no correlation of MeBT results with the number of GAA repeats or their interaction. Thus, measuring of hepatic mitochondrial activity by MeBT does not seem to be a good biomarker on the follow-up of natural history of the disease and the response to drugs in clinical trials.

One limitation of our study is that an influence of co-commitant medication on the MeBT outcome cannot be completely excluded. However, no differences were observed between drug-free FRDA patients (n = 6) and patients on medication (n = 10) and an impairment of mitochondrial metabolism is not described for the listed drugs [11,12,14].

## 5. Conclusions

Taken together, the data of our pilot study using the MeBT in FRDA indicate that FRDA patients exhale significantly smaller amounts of ^13^CO_2 _compared with healthy controls indicating a subclinical liver affection in FRDA.

## Competing interests

The authors declare that they have no competing interests.

## Authors' contributions

Stüwe 1C, 2B, 3A; Goetze 2A, 2B, 2C, 3B; Banasch 1C, 3B; Arning 1C, 2C, 3B; Schmidt 1A, 2C, 3B; Schöls 1B, 2A, 2C, 3B; Saft 1A, 1B, 1C, 2A, 2C, 3A, 3B

1. Research project: A. Conception, B. Organization, C. Execution;

2. Statistical Analysis: A. Design, B. Execution, C. Review and Critique;

3. Manuscript: A. Writing of the first draft, B. Review and Critique;

All authors read and approved the final manuscript.

## Pre-publication history

The pre-publication history for this paper can be accessed here:

http://www.biomedcentral.com/1471-2377/11/145/prepub

## References

[B1] Campuzano V Montermini L Molto MD Pianese L Cossee M Cavalcanti F Monros E Rodius F Duclos F Monticelli A Friedreich's ataxia: autosomal recessive disease caused by an intronic GAA triplet repeat expansion Science New York, NY 1996 271 5254 1423 1427 10.1126/science.271.5254.1423 8596916

[B2] Rotig A de Lonlay P Chretien D Foury F Koenig M Sidi D Munnich A Rustin P Aconitase and mitochondrial iron-sulphur protein deficiency in Friedreich ataxia Nat Genet 1997 17 2 215 217 10.1038/ng1097-215 9326946

[B3] Koutnikova H Campuzano V Foury F Dolle P Cazzalini O Koenig M Studies of human, mouse and yeast homologues indicate a mitochondrial function for frataxin Nature genetics 1997 16 4 345 351 10.1038/ng0897-345 9241270

[B4] Schulz JB Dehmer T Schols L Mende H Hardt C Vorgerd M Burk K Matson W Dichgans J Beal MF Oxidative stress in patients with Friedreich ataxia Neurology 2000 55 11 1719 1721 11113228 10.1212/wnl.55.11.1719

[B5] Filla A De Michele G Cavalcanti F Pianese L Monticelli A Campanella G Cocozza S The relationship between trinucleotide (GAA) repeat length and clinical features in Friedreich ataxia American journal of human genetics 1996 59 3 554 560 8751856 PMC1914893

[B6] Schols L Amoiridis G Przuntek H Frank G Epplen JT Epplen C Friedreich's ataxia. Revision of the phenotype according to molecular genetics Brain 1997 120 Pt 12 2131 2140 9448568 10.1093/brain/120.12.2131

[B7] Schmitz-Hubsch T du Montcel ST Baliko L Berciano J Boesch S Depondt C Giunti P Globas C Infante J Kang JS Scale for the assessment and rating of ataxia: development of a new clinical scale Neurology 2006 66 11 1717 1720 10.1212/01.wnl.0000219042.60538.92 16769946

[B8] Vorgerd M Schols L Hardt C Ristow M Epplen JT Zange J Mitochondrial impairment of human muscle in Friedreich ataxia in vivo Neuromuscul Disord 2000 10 6 430 435 10.1016/S0960-8966(00)00108-5 10899450

[B9] Della Nave R Ginestroni A Giannelli M Tessa C Salvatore E Salvi F Dotti MT De Michele G Piacentini S Mascalchi M Brain structural damage in Friedreich's ataxia Journal of neurology, neurosurgery, and psychiatry 2008 79 1 82 85 10.1136/jnnp.2007.124297 17634216

[B10] Milazzo L Piazza M Sangaletti O Gatti N Cappelletti A Adorni F Antinori S Galli M Moroni M Riva A [13C]Methionine breath test: a novel method to detect antiretroviral drug-related mitochondrial toxicity J Antimicrob Chemother 2005 55 1 84 89 15590719 10.1093/jac/dkh497

[B11] Armuzzi A Candelli M Zocco MA Andreoli A De Lorenzo A Nista EC Miele L Cremonini F Cazzato IA Grieco A Review article: breath testing for human liver function assessment Aliment Pharmacol Ther 2002 16 12 1977 1996 10.1046/j.1365-2036.2002.01374.x 12452932

[B12] Banasch M Emminghaus R Ellrichmann M Schmidt WE Goetze O Longitudinal effects of hepatitis C virus treatment on hepatic mitochondrial dysfunction assessed by C-methionine breath test Aliment Pharmacol Ther 2008 28 4 443 449 10.1111/j.1365-2036.2008.03745.x 18513202

[B13] Banasch M Frank J Serova K Knyhala K Kollar S Potthoff A Brockmeyer NH Goetze O Impact of antiretroviral treatment on (13)C-methionine metabolism as a marker of hepatic mitochondrial function: a longitudinal study HIV Med 2010 10.1111/j.1468-1293.2010.00847.x 20500232

[B14] Wutzke KD Forberger A Wigger M Effect of alcohol consumption on the liver detoxication capacity as measured by [13C]methacetin- and [methyl-13C]methionine-breath tests Isotopes Environ Health Stud 2008 44 2 219 226 10.1080/10256010802066372 18569193

[B15] Banasch M Knyhala K Kollar S Serova K Potthoff A Schlottmann R Schmidt WE Brockmeyer NH Goetze O Disease- and treatment-related predictors of hepatic mitochondrial dysfunction in chronic HIV infection assessed by non-invasive (13)C-methionine breath test diagnostic Eur J Med Res 2008 13 9 401 408 18948231

[B16] Bradley JL Blake JC Chamberlain S Thomas PK Cooper JM Schapira AH Clinical, biochemical and molecular genetic correlations in Friedreich's ataxia Human molecular genetics 2000 9 2 275 282 10.1093/hmg/9.2.275 10607838

[B17] Kam PC Cardone D Propofol infusion syndrome Anaesthesia 2007 62 7 690 701 10.1111/j.1365-2044.2007.05055.x 17567345

[B18] Konig SA Schenk M Sick C Holm E Heubner C Weiss A Konig I Hehlmann R Fatal liver failure associated with valproate therapy in a patient with Friedreich's disease: review of valproate hepatotoxicity in adults Epilepsia 1999 40 7 1036 1040 10.1111/j.1528-1157.1999.tb00814.x 10403231

[B19] Delatycki MB Paris DB Gardner RJ Nicholson GA Nassif N Storey E MacMillan JC Collins V Williamson R Forrest SM Clinical and genetic study of Friedreich ataxia in an Australian population American journal of medical genetics 1999 87 2 168 174 10.1002/(SICI)1096-8628(19991119)87:2<168::AID-AJMG8>3.0.CO;2-2 10533031

